# My Workplace Is Not a Safe Place: Transgressive Behavior and Workplace Harassment in Veterinary Clinics in the Netherlands

**DOI:** 10.3390/vetsci12090870

**Published:** 2025-09-08

**Authors:** Jolanda Jansen, Quintie Stoel, Theo J. G. M. Lam

**Affiliations:** 1 St. Anna Advies, Hogewaldstraat 1B, 6641 KD Beuningen, The Netherlands; quintie@anna-advies.nl; 2 Royal GD, P.O. Box 9, 7400 AA Deventer, The Netherlands; t.j.g.m.lam@uu.nl; 3 Department Population Health Sciences, Faculty of Veterinary Medicine, Utrecht University, P.O. Box 80125, 3508 TC Utrecht, The Netherlands

**Keywords:** veterinary profession, psychological safety, incivility, workplace harassment, client aggression, bullying, discrimination, sexual harassment, mental health, workplace violence

## Abstract

Workplace harassment and transgressive behavior—including aggression, bullying, sexual harassment, and discrimination—is a growing concern in society, including veterinary practice, worldwide. This study explores its prevalence in Dutch veterinary clinics serving different animal species, using an anonymous online survey conducted between December 2023 and January 2024. In total, 632 responses were included in the analyses. The respondents were grouped into veterinary support staff, i.e., nurses, technicians, and receptionists (54.1%), veterinarians (35.1%), practice managers (9.7%), and other (1.1%). The respondents mostly identified themselves as female (94.0%). The findings reveal that 69.6% of respondents experienced at least one form of transgressive behavior in 2023. Aggression was the most common (59.7%), followed by bullying (35.8%), discrimination (14.1%), and sexual harassment (5.9%). Younger professionals (aged ≤ 30) and veterinary support staff reported significantly more aggression and bullying than other groups (*p* < 0.005). Client-related incidents dominated aggression and bullying (*p* < 0.001), while colleagues and supervisors together accounted for most discrimination (*p* < 0.001). The results underscore an urgent need for interventions and cultural change in the veterinary sector.

## Simple Summary

Veterinary professionals face various forms of transgressive behavior and workplace harassment. This article analyses a Dutch survey of 632 veterinary clinic employees, revealing a high prevalence of aggression (59.7%), bullying (35.8%), discrimination (14.1%), and sexual harassment (5.9%). Younger professionals, women, and support staff are especially at risk. The international literature confirms similar patterns globally. Compared to other professions such as human healthcare and academia, veterinary clinics demonstrate higher incidence rates, emphasizing an urgent need for cultural and structural reform. Recommendations include policy change, client education, and workplace interventions.

## 1. Introduction

Recent studies have shown that mental health morbidity among veterinary professionals is high [1,2,3,4,5,6,7,8,9,10,11] and can even lead to burnout [1,12,13,14,15,16,17], early departure from the profession [18,19,20,21,22,23,24,25,26,27], or, in extreme cases, suicide [28,29,30,31,32]. Workplace harassment, incivility of clients and colleagues, and transgressive behavior—both with clients and internally within the team or with supervisors—are frequently cited as contributing factors [1,6,14,33,34,35,36,37,38,39,40,41,42,43,44]. Transgressive behavior is a broad concept. In this study, we use the following definition of transgressive behavior in the workplace environment: “Transgressive behavior is any action being intentional or unintentional, that violates personal, emotional, or professional boundaries, causing discomfort, harm, or an unsafe environment for which no consent has been given” [45,46,47,48,49]. This includes aggression, incivility, bullying, sexual harassment, discrimination, or any other possible behavior that fits this definition.

Transgressive behavior includes many forms of misconduct, misbehavior, or workplace harassment. Experiencing transgressive behavior in the workplace affects workplace safety and mental health. Employers are legally required to implement policies to prevent or mitigate physical and psychological strain and create a safe workplace as stipulated in the Dutch Working Conditions Act (Arbowet) [50]. Other EU countries have also implemented legislation on this aspect. Austria, Denmark, Belgium, several states in Australia, and some provinces in Canada have workplace health and safety laws that explicitly cover bullying and psychological harassment.

Workplace safety in the veterinary clinic has been investigated in several countries, showing that transgressive behavior is prevalent [37,42,43,44,51,52,53,54,55,56,57,58]. In a 2023 survey of the Federation of Veterinarians in Europe, 33% of European vets reported an increase in bullying/harassment and conflicts with clients since the COVID-19 pandemic. This was considered a specific challenge for the future by 9% of the European veterinarians [10].

In 2022, the Dutch Central Bureau of Statistics (CBS) conducted a study on transgressive behavior in various professions [59,60,61]. However, the prevalence of such behavior among veterinary professionals in the Netherlands was not part of that study. Earlier research by Barbonis and Endenburg in 2007 [60] found that 26% of veterinarians and 29% of veterinary assistants had experienced aggressive behavior from clients. A study by the Royal Dutch Veterinary Association showed that 70% of veterinarians reported stress due to price-related discussions with clients, and 33% considered leaving the profession as a result of that [62]. Another survey among veterinary nurses revealed that one in five considered leaving the profession in the near future, citing poor communication with supervisors and clients, as well as a lack of recognition and appreciation from both as key reasons [25,63]. This suggests that, also in the Netherlands, transgressive behavior is indeed present to a significant extent within the veterinary field. At the start of this study, in 2023, there was no up-to-date data on the prevalence of transgressive behavior among veterinary professionals in the Netherlands (including veterinarians, veterinary nurses, assistants, receptionists, and practice managers).

The objective of this study was to determine the extent to which veterinary professionals experienced transgressive behavior—defined as aggression, bullying, sexual harassment, or discrimination—at work in 2023. The study specifically investigates differences based on role (veterinarian, veterinary nurse, supervisor/practice manager), gender identity (male, female, other), age, location, type of animals they work with (companion animals, farm animals, equine), and clinic type (independent or corporate chain).

## 2. Participants and Methods

### 2.1. Online Survey

An online survey was designed to explore the prevalence of transgressive behavior (In Dutch: grensoverschrijdend gedrag) in Dutch veterinary clinics serving different animal species.

For the purposes of this research, transgressive behavior was defined into four categories to participants as encompassing:

1. Physical or verbal violence and/or intimidation, such as insults, abusive language, hurtful remarks, aggression, or physical threats or attacks (Aggression);
2. Bullying, including humiliation, contemptuous or belittling comments, exclusion, ignoring, spreading demeaning messages or images, setting someone up to fail, malicious gossiping (i.e., negative talk about absent colleagues) (Bullying);
3. Sexual harassment, including inappropriate touching, sexually suggestive comments, unwanted sexual invitations, voyeurism or leering, or any other non-consensual physical contact (Sexual harassment);
4. Discrimination, referring to treating people differently, disadvantaging, or excluding them based on (personal) characteristics (Discrimination).

In the questionnaire, the respondents were asked whether they had personally experienced any of these four forms of transgressive behavior in their work during the previous 12 months (2023) (yes/no for each category) and were given the option to add additional remarks (not mandatory). Additional questions covered the perceived source of the behavior (clients, colleagues, or other). If the instigator was reported as ‘other’, a textbox appeared asking for further clarification. In the analyses, ‘other’ was further classified. Finally, the survey collected demographic and work-related information. The survey was estimated to take about five minutes to complete. See Appendix A for the full translated questionnaire.

To ensure confidentiality and to minimize social desirability bias in reporting, the questionnaire was anonymous and did not collect identifiable personal or clinic information. No sensitive personal data were collected. The study was exploratory and did not involve hypothesis-driven interventions; it adhered to Dutch standard ethical practices for survey research. Informed consent was implied by voluntary participation after participants had read the introduction text clearly stating the goal of the study and how the results would be analyzed and reported. No formal ethical approval was sought for this study, as it did not fall under the scope of the Dutch Medical Research Involving Human Subjects Act (WMO). Participation was fully voluntary and anonymous, and the respondents had the option to discontinue the survey at any time.

### 2.2. Survey Distribution and Sample

This study was based on a convenience sample of veterinary professionals working in clinics serving different animal species in the Netherlands. The target population included veterinarians as well as support staff such as practice managers, veterinary technicians, nurses, receptionists, and assistants. Because no centralized registry was available for direct sampling, the survey was disseminated through online professional networks and open participation. The questionnaire (in Dutch) was publicized via veterinary professional groups on social media (e.g., in a Facebook group for practice managers and veterinarians and via LinkedIn profiles of the authors and affiliated organizations) and through newsletters of industry organizations, such as Dactari—a cooperative of veterinary professionals supporting veterinary practices in the Netherlands—and St Anna Advies—a veterinary consultancy agency. Participation was voluntary and anonymous. For that reason, the participants did not receive unique individual codes for completing the survey, which means that multiple participation by the same individual, as well as participation of people younger than 18 year old, could not be entirely ruled out.

### 2.3. Measures and Definitions

For each of the four categories of transgressive behavior, a respondent was counted as having “experienced” that behavior in 2023 if they answered affirmatively to the relevant question. Even a single incident qualified, in line with prior workplace violence surveys that measure one-year prevalence in the Netherlands [59,60,61,64]. The survey did not explicitly distinguish between physical and verbal aggression and intimidation in the prevalence question; instead, they were encompassed under aggression. The survey also asked those who had experienced each type of transgressive behavior to identify who the perpetrator was (allowing multiple choices if applicable): clients, colleagues (coworkers of equal rank), or other (elaborate). Responses on ‘other’ were further classified, including practice owners or senior vets in a supervisory position, among others. These responses were used to calculate what proportion of incidents in each category were attributed to clients vs. colleagues. In presenting the Dutch survey results in this paper, we have translated the findings from Dutch to English using ChatGPT4o. The translations were checked and, where required, corrected by the authors.

### 2.4. Data Analysis

The participants were excluded from the analyses if they had not worked in a Dutch veterinary clinic in 2023. Respondents aged 70 and above were also excluded from the analysis, because the current average retirement age in the Netherlands is <70 years. Descriptive statistics (proportions) were used to summarize the one-year prevalence of each form of transgressive behavior overall and within subgroups (by position (role), gender, age category, province, clinic type (independent or corporate chain), and type of animal). Participants in supporting roles, not being veterinarians or managers, were grouped together under ‘veterinary support staff’, as in the Netherlands, veterinary nurses, technicians, and receptionists are not distinguished. In the Netherlands, most receptionists in veterinary clinics, although not required, are formally trained as ‘paraveterinary assistants’. Their professional qualification enables them to assist in surgeries and provide clinical care, while they also frequently—and in some cases predominantly—perform front-desk tasks as part of their role.

Each survey question included an open text box in which the respondents could elaborate on their experiences. These free-text responses were not systematically analyzed; rather, a selection of illustrative quotes was included in this manuscript to descriptively support the quantitative findings.

Chi-square (Χ^2^) tests were conducted to examine differences between groups; the statistically significant findings (at *p* < 0.05) are reported. For example, age group differences were tested by comparing the youngest and oldest groups where sample sizes were sufficient (specifically 21–30-year-olds vs. 51–60-year-olds). Descriptive statistics were generated in Microsoft Excel v16.82; chi-square tests explored associations using JASP v0.18.3.

## 3. Results

### 3.1. Respondents

In total, 649 completed surveys were received. Based on recorded start and completion times, the survey took, on average, less than two minutes to complete. Seventeen respondents were excluded from the analysis. These exclusions consisted of participants who indicated that they were working outside the Netherlands (ten from Belgium, two from Spain, and one from Germany), one respondent who reported no longer working in the veterinary field, and three respondents who were over the age of 70. Thus, in total, 632 respondents were included in this study.

A full overview of respondent demographics is provided in Table 1. Of these respondents, 94.0% (*n* = 594) identified themselves as female, 5.7% as male (*n* = 36), and 0.3% preferred not to disclose their gender (*n* = 2). Reported ages ranged from under 20 to 70, with the largest age groups being 21–30 (35.4%, *n* = 224) and 31–40 (33.7%, *n* = 213); fewer senior practitioners responded (only ~11%, *n* = 73 were over 50).

With respect to professional roles, 54.1% (*n* = 342) of the respondents reported to be employed in veterinary support positions. Of the participants, 35.1% (*n* = 222) mentioned working as veterinarians, and 9.7% (*n* = 61) reported to work in a managerial or supervisory position. Other functions included facilitatory functions like cleaning or accounting (1.1%, *n* = 7). The type of clinic was approximately equally distributed, with 48.7% (*n* = 308) reported working for a veterinary chain and 46.0% (*n* = 291) for an independent practice.

The majority of the respondents (90.3%, *n* = 571) reported to work with companion animals, followed by 4.3% (*n* = 27) with farm animals, and 2.9% (*n* = 17) with horses. The sample included respondents from every province in the Netherlands, with most participants reporting to work in Zuid-Holland (17.2%, *n* = 109) and Noord-Brabant (17.1%, *n* = 108).

### 3.2. Prevalence of Transgressive Behaviors in Dutch Veterinary Clinics in 2023

Table 2 summarizes the reported prevalence of each specific type of transgressive behavior, both overall and broken down by professional role, gender, type of clinic, and animal species treated.

Out of the 632 respondents, 440 individuals (69.6%) reported to have experienced at least one form of transgressive behavior in the workplace during 2023, while 30.4% reported none of these incidents in the previous year. This overall prevalence indicates that almost 2 in 3 veterinary clinic workers had reported to been subjected to some kind of aggression, bullying, sexual harassment, or discrimination in the previous 12 months.

Aggression was the most commonly reported type of transgressive behavior (59.7%, *n* = 377), followed by bullying (35.8%, *n* = 226). About 1 in 7 (14.1%) experienced discrimination, and approximately 1 in 17 (5.9%) experienced sexual harassment.

As part of the survey, respondents were invited to elaborate on their answer. In total, 366 remarks were submitted in this textbox. Below is a selection of illustrative quotes for each category of transgressive behavior.

**Aggression:**

“I was verbally abused and intimidated during a consultation and threatened with being defamed on social media.”

“A client threatened me and my family over the phone after the death of a dog.”

“The man wanted to smash everything. He refused to leave, so we had to call the police.”

**Bullying:**

“The supervisor mocks absent colleagues behind their backs and even curses at them.”

“A partner in the practice verbally abused and humiliated me. I reported it to the other partners, but the incident was downplayed.”

“My manager clearly disliked me. I was only given unpleasant tasks and never allowed to learn or assist. She spoke to the veterinarians behind my back, and some of them started refusing to work with me, saying ‘not with her’.”

**Sexual harassment:**

“A client kissed my hand while I was restraining a cat for a blood test.”

“I was grabbed by my buttocks.”

“During an evening shift: comments about my breasts — whether they were ‘real’.”

**Discrimination:**

“Because I’m a woman, I’m not welcome at one of our dairy farm clients to assist with cesareans.”

“I’m a foreigner and not yet very fluent in Dutch. Some colleagues refused to speak to me.”

“Unequal pay (20% difference) for the same role and experience.”

#### 3.2.1. Differences by Role and Function

The data reveal differences in reported transgressive behavior by job role within the clinic (Table 2). Veterinary support staff reported the highest prevalence of one or more types of transgressive behavior (73.4%, *n* = 251), followed by veterinarians (67.1%, *n* = 149) and managers (59.0%, *n* = 36). Veterinary support staff mentioned aggression more often than veterinarians (64.6% vs. 54.1%; χ^2^ = 6.29, *p* = 0.012). The difference in reported bullying was not statistically significant between veterinary support staff and veterinarians (38.0% vs. 34.7%, χ^2^ = 0.64, *p* = 0.423). For veterinary support staff and veterinarians, similar levels of sexual harassment (6.4%, respectively, 6.3% χ^2^ = 4.0^10−3^, *p* = 0.952) and discrimination (both 14.9% χ^2^ = 2.3^10−4^, *p* = 0.988) were reported.

#### 3.2.2. Differences by Gender

Of the 594 female respondents, 419 (70.5%) reported experiencing one or more forms of transgressive behavior in the workplace (Table 2). Among the 36 male respondents, 19 (52.8%) reported experiencing one or more forms of such behavior. Both respondents who did not disclose their gender reported having experienced some form of transgressive behavior in 2023. A significant difference was found between male and female respondents, with women (70.5%) reporting transgressive behavior more frequently than men (52.8%) (χ^2^ = 5.053, *p* = 0.025).

#### 3.2.3. Differences by Age

Table 2 shows the difference in reported transgressive behavior per age group of veterinary professionals. Based on the submitted survey results, younger respondents report more transgressive behavior than older respondents. All nine respondents under age 20 mentioned to have experienced at least one type of transgressive behavior in 2023. Veterinary professionals aged 21–30 (*n* = 224) reported significantly more transgressive behavior (χ^2^ = 26.890, *p* < 0.001.) than the ones aged 51–60 (*n* = 59, 80.8% vs. 57.5%) and specifically more incidents of aggression (70.5%) compared to those aged 51–60 (35.6%) (χ^2^ = 24.53, *p* < 0.001). In addition, the age group 21–30 reported significantly more bullying than the age group 51–60 (43.8% vs. 23.7%) (χ^2^ = 7.83, *p* = 0.005). Finally, 10.3% of professionals aged 21–30 reported experiencing sexual harassment, compared to 0.0% in the 51–60 age group (χ^2^ = 6.59, *p* = 0.010).

#### 3.2.4. Differences by Type of Clinic and Animal Species

The prevalence of reported transgressive behavior was 68.8% in chain-affiliated practices and 70.8% in independent practices, with no statistically significant difference between them (χ^2^ = 0.272, *p* = 0.602). No significant differences were found for the specific types of behavior either (Table 2).

Respondents working in equine clinics reported the highest rate of transgressive behavior in 2023 (76.6%, *n* = 17), followed by those in companion animal clinics (70.4%, *n* = 571) and farm animal practices (63.0%, *n* = 27). Although there appears to be a numerical difference in the prevalence of reported transgressive behavior among staff working with different animal types, this difference was not statistically significant (e.g., equine clinics compared to farm animal practices, χ^2^ = 0.877, *p* = 0.349).

#### 3.2.5. Differences by Type of Instigator

Analysis of the open responses from participants who reported ‘other’ as the instigator of transgressive behavior revealed that all such instigators were staff members in supervisory positions, including practice managers, clinical directors, or team leaders. For analysis, these were grouped under the category ‘supervisor’.

Among all participants who reported experiencing aggression, the majority attributed it to clients (78.7%, *n* = 328), with a smaller share involving colleagues and supervisors (21.3%, *n* = 89), a difference that was highly significant (χ^2^ = 77.9, *p* < 0.001; Table 3). Bullying was also significantly more often reported as caused by clients (55.6%, *n* = 84, clients vs. 44.2%, *n* = 62, colleagues, χ^2^= 14.9, *p* < 0.001). Sexual harassment was reported somewhat more often from clients (60.5%, *n* = 23) than from team members (39.5%, *n* = 13), although this difference was not statistically significant (χ^2^ = 0.54, *p* = 0.46). Discrimination, by contrast, was most frequently reported as originating from within the respondent’s own team (72.3% in total: 52.5% colleagues (*n* = 53), 19.8% supervisors (*n* = 20), significantly more often than from clients (27.5%, *n* = 28; χ^2^ = 56.2, *p* < 0.001). 

## 4. Discussion

This study found that 70% of veterinary professionals in the Netherlands reported some form of transgressive behavior in their workplace in 2023. This equates to more than two in every three employees in veterinary clinics. Of the four categories of transgressive behavior used in this study, verbal or physical aggression, including intimidation, was the most frequently reported (60%), followed by bullying (36%), discrimination (14%), and sexual harassment (6%). Veterinary support staff (e.g., veterinary nurses, assistants, technicians, and receptionists) in this study reported more aggression (65%) than veterinarians (54%). This trend mirrors findings from a 2007 Dutch study [60], although the overall reported prevalence of aggression seemed lower at that time, 29% in 2023 vs. 26% in 2007. This suggests an increase in transgressive behavior in Dutch veterinary clinics over time. In a 2023 survey of the Federation of Veterinarians in Europe, 33% of European vets reported an increase in bullying/harassment and conflicts with clients since the COVID-19 pandemic. In that study, 9% of the European veterinarians considered this as a challenge for the future [10].

Internationally, several studies have also been conducted on themes like workplace harassment, misconduct, discrimination, bullying, and incivility, using different research designs. A 2022 UK/Ireland survey of 252 veterinary staff also revealed incivility from clients, colleagues, and senior staff, with the latter significantly associated with burnout, poor mental health, reduced job satisfaction, and increased intentions to leave the profession [44]. In 2021, the British Veterinary Association [65] found that 15% of veterinarians reported to have had personally experienced discrimination, and 21% had witnessed it. In a 2019 UK-based RCVS survey, it was found that 96% of students and early-career veterinary nurses considered bullying and incivility to be concerning issues in their work [54]. Also, a UK study in 2025 found that racism is a serious concern in veterinary clinics [66], with 36% of British/Irish veterinary professionals reporting discrimination during extra-mural studies [67]. Similarly, a study from New Zealand in 2022 reported that 70% of veterinary nurses had personally experienced bullying by staff, while 81% mentioned that they had witnessed such behavior in their clinic [68]. Furthermore, 16% of New Zealand veterinarians reported to have been bullied at work according to a 2018 study [69]. These studies indicate that the findings of this Dutch study are consistent with international experiences [4,10,14,21,42,44,48,53,66,69,70,71,72,73,74,75] and that workplace misconduct and transgressive behavior is a global issue in veterinary medicine.

Veterinary staff—especially those in client-facing roles—are more exposed to transgressive behavior, as previously shown in other studies [44,52,54,73,75,76]. No statistically significant differences were observed between respondents serving different types of animal species, although it has to be noted that the number of participants from livestock and equine clinics was limited. There is a need for longitudinal studies, studies including the different types of clinics, different roles of veterinary professionals, and repetition of studies in other countries to confirm the current status of transgressive behavior and workplace misconduct and the possible increase in the prevalence in veterinary clinics over time.

Veterinary professionals in our study reported substantially more misconduct than professionals in comparable sectors in the Netherlands. According to the Netherlands Working Conditions Survey [59,61], 11% of workers in the general Dutch workforce reported to have experienced aggression, 5% bullying, 10% discrimination, and 4% sexual harassment (Table 4). This is lower than the prevalences found in our study and in the previously mentioned comparable studies in veterinary clinics in other countries. Research from Australia, however, shows that veterinary students reported higher levels of psychological distress compared to the average Australian population [77].

Professionals in the medical sector in the Netherlands also reported lower rates of transgressive behavior than veterinary professionals. In a study among human healthcare providers similar to ours, executed and published by Medisch Contact, one of the leading professional journals for medical professionals in the Netherlands, 52% of physicians and medical students reported having ever experienced one or more forms of transgressive behavior in 2023 [64]. In contrast, 70% of veterinary professionals in the present study reported this in the same one-year period. More specifically, in the human health study, all forms of transgressive behavior, besides discrimination, were reported less frequently, with 19% experiencing aggression or bullying in the previous year, and 13% reporting sexual misconduct [64]. Research from Australia also indicates that veterinary students experience higher levels of psychological distress than their medical counterparts [77]. Factors influencing transgressive behavior, like team hierarchy or working under high stress levels [43,44,48,56,57,58,70,78], may be similar in human and veterinary medicine. Nevertheless, Dutch veterinary professionals appear to face more frequent aggression and bullying.

Transgressive behavior in the human healthcare was mainly caused by medical specialists and supervisors with above 60% causing at least one of all forms of transgressive behavior [64]; meanwhile, in this study, most instigators were clients, except for discrimination. Part of the difference between veterinary and human medicine regarding transgressive behavior caused by clients could be explained by increasing veterinary costs and financial hardship among pet owners. This may increase tensions and trigger aggression towards the veterinary staff [57,62]. Poor communication between staff and management—as well as between veterinary staff and clients—can further exacerbate tensions, particularly in high-stakes situations such as medical emergencies, financial constraints, or euthanasia discussions [17,43,56,79]. The structure of veterinary clinics may also play a role where conflict management protocols sometimes are absent or informal [1,38,43,80] compared to human medicine hospitals. When combined with the limited communication training provided in veterinary curricula, these structural and interpersonal factors may contribute to situations such as unmet client expectations or friction between colleagues, which in turn can foster incivility and transgressive behavior [14,23,43,44,56,69,79,81,82,83]. However, more research is needed to investigate and confirm the possible difference between veterinary and human medicine and to understand why veterinary professionals experience more transgressive behavior than their human medicine counterparts.

Younger professionals in this study reported more transgressive behavior across all categories, which is consistent with findings in other sectors and studies in veterinary clinics from other countries [40,42,44,57,67,77,84,85,86]. For instance, younger age has been associated with higher vulnerability to workplace incivility and harassment in healthcare and service industries [44,87].

Gender differences were observed, with women reporting more transgressive behavior than men, and younger respondents also reporting it more frequently, consistent with the broader literature on gender, age, psychological distress, and workplace misconduct [1,11,51,52,69,77,86,87,88,89,90,91]. The high proportion of women participants in our study (94%) limits generalizability of these results for how other genders perceive transgressive behavior in the veterinary clinic. It reflects, however, the future gender distribution in the field, with 86% of current graduating veterinary students in the Netherlands being female [25,91] and global trends showing a feminization of the veterinary workforce [10,91]. No specific national data is available of veterinary support staff, but women are also overrepresented in these functions.

There is an ongoing discussion on how different generations, genders, nationalities, and neurodivergent and neurotypical people perceive transgressive behavior and workplace-related stress—i.e., what is perceived as transgressive by one person could be perceived as humor by another—and differences exist about what is perceived as acceptable in a workplace environment [7,29,45,47,56,58,66,67,85,92,93,94,95,96,97,98,99,100,101,102,103,104,105]. Given the current literature on this topic and the findings of this study, there is not only a need to better prepare the next generations of veterinary professionals for the challenges ahead but also a broader need for cultural change within the veterinary profession.

While the prevalence rates reported in this study are concerning and indicative of a widespread issue, several limitations should be acknowledged. First, the sample size is limited, and a larger number of responses would undoubtedly have provided a more comprehensive picture of psychological safety within the veterinary profession. Nevertheless, the current study provides a sizeable convenience sample, estimated to approximately represent 5–10% of the active veterinary clinic workforce in the Netherlands [23]. This proportion indicates that, despite its limitations, the dataset offers valuable insights into the experiences of a meaningful segment of the profession.

A voluntary response bias may have influenced the sample, as individuals who experienced workplace misconduct might have been more inclined to participate. To mitigate this, the survey explicitly invited responses from all veterinary professionals, regardless of their experiences with transgressive behavior. Nevertheless, the results of this study could be an overestimation of the experiences of all veterinary professionals. Participants did not receive unique identifiers, so the possibility of multiple participation cannot be excluded. Additionally, social desirability bias may have led to underreporting, particularly with respect to peer-to-peer misconduct, due to the fact that participants could respond anonymously, so they could have been afraid to share the real situation out of shame or fear of retaliation. Future studies should ensure that participants are provided with appropriate resources for support before, during, and after the completion of the survey.

Recall bias could have affected the accuracy of retrospective self-reports. The composition of the sample—characterized by an overrepresentation of women and people working with companion animals—may limit generalizability on how male veterinary professionals and people working with livestock and equine experience transgressive behavior. Nevertheless, this distribution reflects the current demographics of the Dutch veterinary workforce [25]. Moreover, the study’s cross-sectional design restricts causal interpretations and precludes analysis of temporal trends. While attempts were made to define specific categories, there is potential for overlap between response categories; for example, bullying may involve sexual harassment. Therefore, some degree of interpretative overlap between response categories cannot be excluded, like being intimidated because of personal characteristics like gender or nationality. These limitations highlight the need for further longitudinal and mixed-methods research to more deeply explore the underlying dynamics, causal pathways, and long-term consequences of transgressive behavior in veterinary clinics.

The findings of this study support an urgent call for action. Under the Dutch Working Conditions Act (Arbowet), as well as in many other countries worldwide, veterinary employers are legally required to protect their staff from psychosocial work-related hazards. However, to date, no veterinary-specific protocols for addressing workplace misconduct appear to be publicly available. Based on the results of this and previous research, several actions are recommended. While this overview is not exhaustive, it highlights key strategies that are frequently recommended in the literature as suggestions to improve workplace safety in veterinary clinics [14,28,40,43,56,75,78,79,94,95,104,106]. These include developing clear definitions and practical examples of unacceptable behavior—covering clients, colleagues, and supervisors alike—and establishing behavioral codes of conduct for both internal and external stakeholders [47,95]. Staff training in communication and conflict management, particularly for those in client-facing roles, should be prioritized [107]. Young veterinary professionals should receive more support and mentoring [108,109]. Clinics should also provide access to an independent and external confidential advisor or Employee Assistance Program [110], promote open dialog [47], and implement anonymous reporting procedures that protect the rights and privacy of all parties involved. Reports of misconduct, regardless of whether it is reported by the instigator or victim, must be addressed swiftly and seriously, with strong protections in place against retaliation [104,105]. In addition, structured aftercare, including access to mental health support, should be offered [100,111], and all protocols should be integrated into onboarding and ongoing training processes. Finally, veterinary educational institutions are encouraged to embed both preventive and responsive strategies into their curricula to better prepare future professionals for the demands of veterinary practice.

## 5. Conclusions

Transgressive behavior is widespread in Dutch veterinary clinics, surpassing national averages and reflecting global patterns. More than two-thirds of employees in veterinary clinics experienced at least one form of transgressive behavior in 2023. Clients are the primary perpetrators of aggression and bullying, while the internal team accounted for most of the reported discrimination. Young professionals and veterinary support staff are particularly vulnerable to transgressive behavior. Our findings highlight both the importance of preparing future veterinary professionals and the urgent need for cultural change within the profession, underscoring the necessity of targeted interventions.

## Figures and Tables

**Table 1:** Reported descriptive characteristics of veterinary professionals participating a 2023 survey on transgressive behavior in veterinary clinics in the Netherlands (*N* = 632).

Demographics		% of Total (*N* = 632)
Gender	Female	94.0% (594)
	Male	5.7% (36)
	Other	0.3% (2)
Age	<20	1.4% (9)
	21–30	35.4% (224)
	31–40	33.7% (213)
	41–50	17.9% (113)
	51–60	9.3% (59)
	61–70 ^1^	2.2% (14)
Role/Function	Veterinary support staff ^2^	54.1% (342)
	Veterinarian	35.1% (222)
	Manager/team lead	9.7% (61)
	Other	1.1% (7)
Type of clinic	Corporate chain	48.7% (308)
	Independent	46.0% (291)
	Not reported	5.2% (33)
Animal species	Companion animals	90.3% (571)
	Livestock	4.3% (27)
	Horses	2.9% (17)
	Mixed	1.9% (12)
	Other	0.8% (5)
Province of the Netherlands	Zuid-Holland	17.2% (109)
	Noord-Brabant	17.1% (108)
	Gelderland	15.0% (95)
	Utrecht	13.8% (87)
	Noord-Holland	13.4% (85)
	Overijssel	5.5% (35)
	Limburg	5.4% (34)
	Friesland	4.7% (30)
	Flevoland	4.4% (16)
	Drenthe	2.4% (15)
	Groningen	1.9% (12)
	Zeeland	1.0% (6)

^1^ Participants > age of 70 years were excluded from the analyses. ^2^ Veterinary nurses, technicians, assistants, and receptionist are grouped together.

**Table 2:** Frequency distribution of reported forms of transgressive behavior among veterinary professionals participating a 2023 survey on transgressive behavior in veterinary clinics in the Netherlands (*N* = 632).

Demographic Category		Aggression	Bullying	Sexual Harassment	Discrimination	Total Transgressive Behavior ^1^
Participants	All veterinary professionals (*N* = 632)	59.7%	35.8%	5.9%	14.1%	69.6%
Role/Function	Veterinary support staff ^2^ (*n* = 342)	64.6%	38.0%	6.4%	14.9%	73.4%
	Veterinarian (*n* = 222)	54.1%	34.7%	6.3%	14.9%	67.1%
	Manager/team lead (*n* = 61)	54.1%	31.1%	0.0%	8.2%	59.0%
	Other (*n* = 7)	42.9%	14.3%	14.3%	0.0%	57.1%
Gender	Female (*n* = 594)	60.3%	36.5%	6.1%	14.3%	70.5%
	Male (*n* = 36)	47.2%	19.4%	0.0%	8.3%	52.8%
	Other (*n* = 2)	100.0%	100.0%	50.0%	50.0%	100.0%
Age	<20 (*n* = 9)	77.8%	66.7%	0.0%	22.2%	100.0%
	21–30 (*n* = 224)	70.5%	43.8%	10.3%	15.2%	80.8%
	31–40 (*n* = 213)	60.6%	38.0%	4.7%	16.0%	70.4%
	41–50 (*n* = 113)	50.4%	22.1%	2.7%	10.6%	58.4%
	51–60 (*n* = 59)	35.6%	23.7%	0.0%	11.9%	57.6%
	61–70 (*n* = 14) ^3^	35.7%	14.3%	7.1%	0.0%	42.9%
Type of clinic	Independent (*n* = 291)	59.1%	36.1%	7.6%	13.1%	70.8%
	Corporate chain (*n* = 308)	61.4%	35.1%	4.2%	14.0%	68.8%
	Not reported (*n* = 33)	48.5%	39.4%	6.1%	24.2%	66.7%
Animal species	Horses (*n* = 17)	64.7%	47.1%	11.8%	5.9%	76.5%
	Companion animals (*n* = 571)	60.8%	35.7%	5.6%	13.7%	70.4%
	Livestock (*n* = 27)	48.2%	33.3%	11.1%	25.9%	63.0%
	Mixed (*n* = 12)	50.0%	25.0%	0.0%	16.7%	50.0%
	Other (*n* = 5)	0.0%	40.0%	0.0%	20.0%	40.0%

^1^ One or more of the four types of transgressive behavior was experienced in 2023. ^2^ Veterinary nurses, technicians, assistants, and receptionist are grouped together. ^3^ Participants > age of 70 years were excluded from the analyses.

**Table 3:** Instigators of transgressive behavior reported by veterinary professionals participating in a survey on transgressive behavior in veterinary clinics in the Netherlands in 2023 (*N* = 632).

		Aggression	Bullying	Sexual Harassment	Discrimination
Caused by	Clients	78.7%	55.6%	60.5%	27.7%
	Colleagues	15.3%	41.1%	34.2%	52.5%
	Other: Supervisors ^1^	6.0%	3.1%	5.3%	19.8%

^1^ Participants who selected ‘other’ specified individuals in supervisory roles (e.g., practice managers, clinical directors, team leaders). These responses were therefore grouped under the category ‘supervisors’.

**Table 4:** Frequency distribution of reported forms of transgressive behavior among other professions and healthcare professionals in the Netherlands in 2023 based on available national data.

		Aggression	Bullying	Sexual Harassment	Discrimination
Veterinary professionals 2023 (our study)	All veterinary professionals (*N* = 632)	59.7%	35.8%	5.9%	14.1%
Healthcare study 2023 ^2^	Doctors and medical students (human medicine) (*N* = 6569)	18.9%	18.9%	12.8%	25.0%
National data 2023 ^1^	General Dutch workforce (*N* = 100)	11.6%	5.3%	4.5%	10.7%
	Doctors (human medicine) (*N* = 100)	27.8%	5.3%	11.8%	11.6%
	Human medical support staff (*N* = 100)	23.7%	6.3%	8.3%	12.4%

^1^ Data based on national statistics in 2023, human medical support staff includes, e.g., nurses in hospitals and pharmacist receptionists. No total scores were available [59,61]. ^2^ Data based on similar online survey in 2023 by Medisch Contact [64].

## Data Availability

The raw data supporting the conclusions of this article will be made available by the authors on request.

## Appendix A. Online Survey Questionnaire

Survey on transgressive behavior in veterinary medicine (translated from Dutch).

There are four main forms of transgressive behavior in the workplace. These can occur both physically and online. For each type, we ask if you have encountered it and by whom.

In the past 12 months, have you encountered any form of physical or verbal violence and/or intimidation (such as insults, swearing, hurtful expressions, aggression, or physical threats or attacks)?

- Yes
- No

By whom did you experience physical or verbal violence and/or intimidation?

- A customer or customers
- A colleague or colleagues
- Other, namely: …

Any additional comments: [Enter your answer] *

In the past 12 months, have you experienced any form of bullying (such as humiliation, derogatory and belittling comments, ignoring and excluding, spreading threatening or degrading messages or photos, sabotaging someone, gossiping, or badmouthing)?

- Yes
- No

If your answer to the previous question was ‘yes,’ by whom did you experience bullying?

- A customer or customers
- A colleague or colleagues
- Other, namely: …

Any additional comments: [Enter your answer] *

In the past 12 months, have you experienced any form of sexual harassment (such as unwanted touching, inappropriate sexual comments, invitations for sexual acts, peeping and winking, or any other physical contact that is not accepted)?

- Yes
- No

If your answer to the previous question was ‘yes,’ by whom did you experience sexual harassment?

- A customer or customers
- A colleague or colleagues
- Other, namely: …

Any additional comments: [Enter your answer] *

In the past 12 months, have you experienced any form of discrimination in the workplace (such as treating people differently, disadvantaging, or excluding them based on (personal) characteristics)?

- Yes
- No

If your answer to the previous question was ‘yes,’ by whom did you experience discrimination?

- A customer or customers
- A colleague or colleagues
- Other, namely: …

Any additional comments: [Enter your answer] *

A few more questions about yourself:

How do you identify yourself?

- Female
- Male
- Prefer not to say
- Other, namely: …

What is your age?

- <20
- 21–30
- 31–40
- 41–50
- 51–60
- 61–70
- >70

In which province do you work? [Enter your answer]

Where do you work?

- Veterinary practice
- Other, namely: …

What type of practice is this?

- Independent clinic
- Part of a chain
- I don’t know

Which chain is this? [Enter your answer] *

What is your position?

- Veterinarian
- Paraveterinary
- Practice manager
- Veterinary assistant
- Other, namely: …

With which animal species do you work the most?

- Companion animals
- Farm animals
- Horses
- Other, namely: …

Do you have any comments, suggestions, or ideas to improve social safety in the workplace? [Enter your answer] *

* This question was not mandatory to answer.
